# Effectiveness of face-to-face physiotherapy training and education for women who are undergoing elective caesarean section: a randomized controlled trial

**DOI:** 10.1186/s40945-021-00128-9

**Published:** 2022-02-03

**Authors:** Kalani Weerasinghe, Mohamed Rishard, Subhani Brabaharan, Aysha Mohamed

**Affiliations:** 1 Department of Allied Health Sciences, Faculty of Medicine, University of Colombo, Kynsey Road, Colombo, 08 Sri Lanka; 2 Department of Obstetrics and Gynecology, Faculty of Medicine, University of Colombo, No 25, Kynsey Road, Colombo, 08 Sri Lanka; 3 National Hospital, Colombo, 10 Sri Lanka; 4 Department of Obstetrics and Gynecology, Faculty of Medicine, University of Colombo, No 25, Kynsey Road, Colombo, 08 Sri Lanka

**Keywords:** Face-to-face physiotherapy training and education, Elective caesarean section, Enhanced post-operative recovery, Prehabilitation

## Abstract

**Background:**

Caesarean Section (CS) is associated with numerous post-operative problems. The current literature reveals that physiotherapy interventions such as pelvic floor rehabilitation and post-surgical rehabilitation enable enhanced recovery in the post-operative period. The purpose of this study was to investigate the effectiveness of face-to-face physiotherapy training and education prior to elective CS in improving post-operative outcomes.

**Methods:**

A single blind parallel randomized controlled study was carried out at De Soysa Hospital for Women (DSHW), Colombo. Fifty-four women who were to undergo elective CS were recruited to the study. The women in the intervention group (*n* = 27) received face-to-face physiotherapy training and education; the control group (n = 27) received only the standard nursing care. Outcome measures such as perception of post-operative pain, dosage of additional analgesics required, pain upon returning to functional activities and lengths of hospital stay were collected. Results were analyzed using IBM SPSS 20 using descriptive statistics and independent samples t-test.

**Results:**

Mean post-operative pain score (control group; 4.2±0.46 vs. intervention group; 1.7±0.7) and doses of additional analgesics required were significantly higher in the control group than that of the intervention group. Pain upon returning to functional activities decreased significantly within 2 days in both groups, and values were lower in the intervention group. The intervention group showed a shorter hospital stay than the control group (control group;3.9 ± 0.3 vs. intervention group;3.00 ± 0.0) (*p* < 0.05).

**Conclusions:**

Face-to-face physiotherapy training and education prior to elective CS appears to be a promising intervention to improve the post-operative outcomes by reducing post-operative pain, doses of additional analgesics required, pain upon returning to functional activities and lengths of hospital stay.

**Trial registration:**

SLCTR/2019/029-APPL/2019/028; Registered on 6th of September 2019.

## Background

Caesarean Section (CS) is one of the most common surgeries performed in obstetric practice. It involves termination of a pregnancy and delivery of the live or dead fetus through an incision in the abdominal wall rather than through the pelvis and vagina [1–3]. The ‘rising tide’ of CS is a grave concern. It is associated with numerous complications, leading to a poor return to functional activities, which have a significant impact on the general health status [4–6]^.^ Incision-related pain, intestinal problems, mastitis, depression, nausea, vomiting and anxiety are some of the problems encountered in the post-operative period [7–13].

Physiotherapy is an essential component of post-partum care [14]. Physiotherapy during the early post-operative period effectively reduces incision-related pain, enables early commencement of functional activities, facilitates ambulation, and return of bowel activity [15]. Mobility exercises, breathing techniques and postural care have been shown to reduce immediate incision-related pain and difficulty in functional activities by the 2^nd^ post-operative day [16]. Individuals who received physical therapy had significantly improved outcomes compared with the standard care group, suggesting that physical therapy may be a helpful adjunct to improve recovery following CS [17]. Reduced pain and early return to functional activities following CS by post-natal exercises such as deep breathing, inter-digital technique for chest expansion, protected huffing technique, ankle pumps, leg sliding, pelvic rolling, and abdominal wall setting were noted, and it was pointed out that post-natal physiotherapy is important in reducing the pain score, difficulty associated with functional activities, time for ambulation, and analgesic intake on the 1^st^ and 2^nd^ post-operative days [16].

The effect of conservative physiotherapy techniques, has shown to reduce post-operative pain, incision related adhesions and diastasis [18]. There is clear evidence that early ambulation is effective in enhancing post-operative recovery following CS [19]. Apart from these benefits, breathing exercises and respiratory physiotherapy have also shown many beneficial effects in improving pulmonary function in females having CS [20, 21]. A recent study by Burti and colleagues reported that physiotherapy exercise protocol optimistically contributed to the reduction of pain and improvement of general well-being [22]. The use of opiate based analgesics for post-operative pain management and hospital stay following surgery was reduced when a proper physiotherapy program was incorporated [8]. Physiotherapy programs in the early post-caesarean  period are effective in a wider perspective than the current literature and are considered valuable for increasing the quality and productivity of post-natal care, therefore improving well-being after childbirth [15]. However, there is a lack of a structured physiotherapy training and education program incorporated as part of post-operative care following CS to improve and enhance post-operative outcomes [23].

A functional decline is frequently associated with major surgery. Prehabilitation is the process of enhancing an individual’s functional capacity in order to enable the patient to withstand a forthcoming stressor. It is a multimodal approach comprised of medical optimization, pre-operative physical exercise, nutritional support and stress/anxiety reduction [24]. It has been shown that educating patients prior to surgery by trained healthcare workers improves patients’ adherence to physiotherapy, which in turn leads to improved post-operative outcomes [25].

However, there are only a limited number of studies available regarding physiotherapy and its impact on enhanced post-natal care, globally. Very few studies have investigated systematically aspects such as optimal timing of physiotherapy education, method of delivery, duration and frequency of reinforcement and feedback.

The primary objective of our study was to investigate the effectiveness of face-to-face physiotherapy training and education prior to elective CS in improving post-operative pain. The secondary objectives included other key post-operative outcomes such as doses of additional analgesics required, pain upon returning to functional activities, and lengths of hospital stay. Hence, this study aims to examine whether face-to-face physiotherapy training and education for women who are undergoing elective CS leads to improvement in post-operative outcomes?

We hypothesized that an interventional program comprised of face-to-face physiotherapy training and education given in the antenatal period,reinforcement in the post-operative period with regular feedback and encouragement would contribute to improved post-operative outcomes, which is a key element of prehabilitation.

## Methods

The CONSORT 2010 statement guidelines regarding randomized trials (www.consort-statement.org) were followed for this RCT [26].

### Trial design

**Fig. 1:**
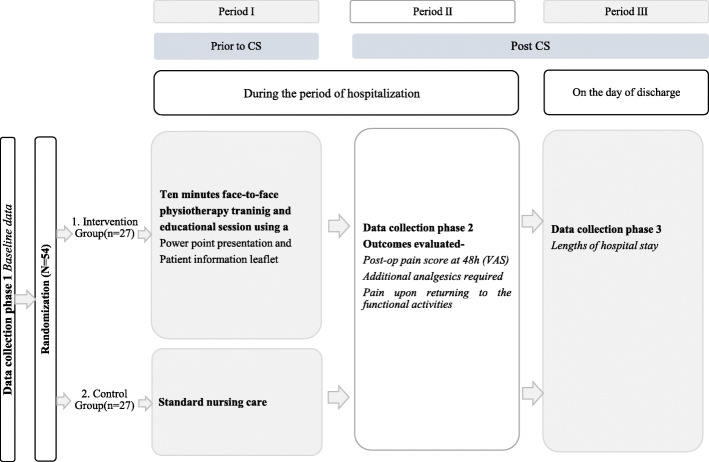
Flow chart of this randomized controlled trial

The study design adopted for this study was a single centre single blind randomized controlled parallel group trial design. This Randomized Controlled Trial (RCT) included 54 women who were randomly allocated to an intervention or a control group. Figure 1 describes the flow of this RCT. This was conducted at the professorial unit, De Soysa Hospital for Women (DSHW), Colombo, which is affiliated to the Faculty of Medicine, University of Colombo, Sri Lanka. Written informed consent was obtained from all study participants upon recruitment to the study and they were given the option to withdraw from the study or follow up without any consequences to their further clinical management in accordance with the Helsinki declaration. Ethical approval for the study was obtained from the Ethics Review Committee of the Faculty of Medicine, University of Colombo (UCP-AL-15-319) and the study was registered at the Sri Lanka Clinical Trials Registry (SLCTR/2019/029-APPL/2019/028). Permission was obtained from the director of DSHW, Colombo, to conduct the study.

### Participants

Eligible women aged 20–40 years who were to undergo elective CS were asked to participate in the study. The inclusion criteria for the study were women who had given informed written consent for Category “4” CS due to fetal and maternal indications. The exclusion criteria were patients who had undergone two or more CSs or abdominal surgeries, who had complications such as Diabetes Mellitus, Systemic Lupus Erythematosus, connective tissue disorders, sepsis, patients on Disease Modifying Anti Rheumatic Drugs (DMARDs) and oral steroids, patients who could not comply with the physiotherapy interventions such as mentally incapacitated patients, patients who were in the Intensive Care Unit (ICU), deliveries with operative complications, CSs with general/epidural anesthesia and using patient-controlled anesthesia, and patients with abdominal hernia and Diastasis Rectus Abdominis (DRA) larger than 2 cm.

### Intervention

#### Intervention group

The participants in the intervention arm were given 10-minute face-to-face physiotherapy training and educational sessions pre-operatively by a trained physiotherapist (K.W.). A PowerPoint presentation was used to educate the participants during this session, and it was supplemented with a patient information leaflet. This presentation consisted of information on how CS is carried out, indications for CS, potential risks and complications associated with CS, the importance of physiotherapy to minimize potential complications following CS, physiotherapy education guidelines, exercise prescription to be followed during the particular time intervals after CS, number of repetitions and frequency of each exercise. The exercise techniques were taught and carried out initially under the supervision of the trained physiotherapist, and feedback was offered on the technique. Participants in the intervention group were advised to perform each exercise twice a day with 10–12 repetitions per session. All the exercises were planned to commence from the day of the surgery until the day of discharge. All exercises and education guidelines adopted were evidence-based and described in Table 1 [15, 16, 27, 28]. An information leaflet with details on the exercise prescription with pictorial representation, repetition, frequency of each exercise, physiotherapy education guidelines, and precautions to be taken during exercises was handed over to the participants as a supplement. Any clarifications with regard to exercises prescribed were addressed. Reinforcement with regard to exercise prescription and education guidelines was carried out twice a day by the research team. Feedback was offered in the post-operative period by the research team to ensure adherence and treatment fidelity after the CS. All measures were taken to minimize the close contact between the intervention group and the control group. Face-to-face physiotherapy sessions were carried out in a private room to reduce potential bias in the post-CS care that they received, and therefore, nurses caring for post-partum women were unaware of the intervention group or the control group. Hence, both groups received the standard nursing care.

**Table 1:** Exercise Prescription and patient education program

Time interval	Plan of care
1.During 1^st^ 24 h	‑ Thoracic expansion exercises; wound support and breathing **(to improve exchange of gases)** ‑ Protected Huffing technique **(to remove secretions)** ‑ Simple leg exercises **(to improve blood circulation and relax calf muscles)**
2. From 1^st^ post-operative day to the day of discharge	‑ Deep breathing exercise for wind pain **(to relieve pain improve exchange of gases)** ‑ Support the incision with a pillow when coughing, moving, or breastfeeding, as well as education on incisional care and injury risk **(to reduce incision-related pain when coughing, moving, or breastfeeding).** ‑ Ankle pumps and leg sliding **(to improve blood circulation and relax calf muscles)** ‑ Pelvic rolling and abdominal wall setting exercise **(to stimulate intestinal activity, contract abdominal muscles and prevent or control gas pain)** ‑ **Patient education regarding postural adjustments, particularly on child care, body mechanics instructions, positioning for activities of daily living, and education on getting in and out of bed (to correct posture and protect activities of daily living).** ‑ Education on risk factors and types of pelvic floor dysfunction **(to prevent pelvic floor dysfunction)**

#### Control group

The control group was only given the standard nursing care, which did not include any physiotherapy training or educational sessions, and was followed up during this time period.

### Outcomes

Outcomes were obtained during three data collection phases: baseline prior to CS (Phase 1), 24-h and 48-h following CS (Phase 2), and discharge day (Phase 3). Baseline data such as age, weight, height, and parity were obtained, and the booking Body Mass Index (BMI) was obtained from the patient’s clinical records.

#### Primary outcomes

The primary outcome of the study was the post-operative pain score at 48 h after CS, which was measured using the Visual Analogue Scale (VAS). Participants were asked to mark their overall severity of pain between 0 (indicating no pain) and 10 (indicating very severe pain). The relevant VAS score was then determined by measuring in millimeters from the left-hand end of the line to the point that the patient marked using a ruler. A higher score indicates greater pain intensity.

#### Secondary outcomes

The secondary outcomes of the study were pain upon returning to functional activities, doses of additional analgesics required, and lengths of hospital stay.

The pain experienced during each of the activities such as turning in bed without support, sitting without support, standing without support and walking without support were recorded to evaluate the pain upon returning to functional activities on the first and second post-operative days. These were measured using the VAS. Doses of additional analgesics required and lengths of hospital stay were obtained from the patients' clinical records on the day of discharge and recorded on the data collection forms.

### Sample size

The required number of study participants for the primary outcome (post-operative pain score at 48-hh) was calculated using the findings from available literature. The expected effect size (mean difference in post-operative pain score) was taken as 1.62 (SD-1.94) [15]. The probability of type I error-alpha was set at 0.05 and the power at 80% [29]. Thus, the calculated sample size was 23 mothers in each arm with a 1:1 ratio. Further, 15% was added to the sample, allowing for non-response. **Therefore, the final sample size in each group was 27 women who were elective for CS.**

### Randomization

Pregnant women who were to undergo elective CS were randomly assigned to two groups, as the intervention group and the control group, using a simple randomization design. To reduce bias in the allocation of the participants to the two arms of the study, a computer-generated random number sequence was placed within opaque, sealed envelopes and used to determine the allocation of participants to each arm upon recruitment to the study.

### Blinding

This was a single blind study where participants were blinded with a definitive intervention. Therefore, there was no attempt made to conceal the identity of each group from the investigators. The same physiotherapist implemented pre-operative interventions and carried out the outcome measurements.

### Statistical methods

Data obtained from the two groups was analyzed using the Statistical Package for the Social Sciences (SPSS 20.0). The significance level was set at 0.05 and calculated at 95% confidence interval. Descriptive statistics were utilized to analyze socio-demographic details between the intervention and control groups. An independent samples t-test was used to compare the post-operative outcomes following CS between the two groups.

## Results

### Participant flow

**Fig. 2:**
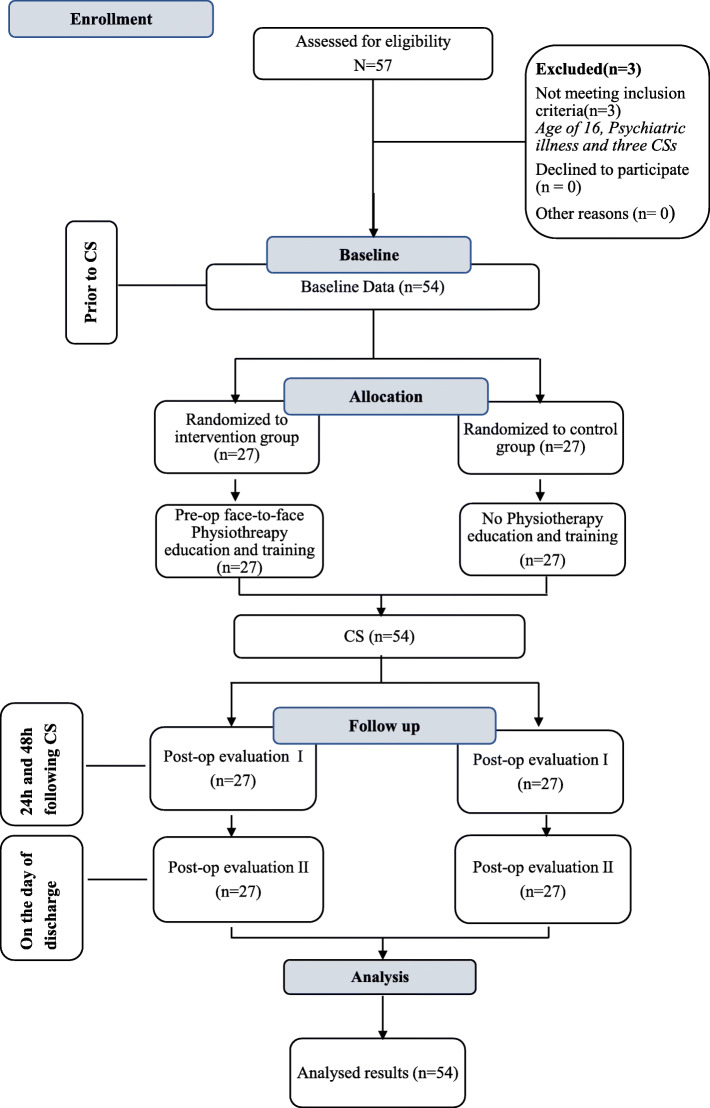
Flow chart of participants

All participants who were recruited to the study were included as part of the final analysis as there were no dropouts from the time of recruitment. The flow of participants through the study is shown in Fig. 2.

### Recruitment

Participants were recruited over a period of 7 months from March 2020 to September 2020, and recruitment of subjects was stopped when the required numbers of study participants were obtained. All participants were followed up until the day of hospital discharge (Fig. 2).

### Baseline data

Age, body weight, height, BMI at the booking visit, and parity were obtained as the baseline data and socio-demographic characteristics of the study population are shown in Table 2.

**Table 2:** Information on socio-demographic characteristics and descriptive data on anthropometry of the study population

Characteristic	Intervention Group n = 27 Mean ± SD	Control Group *n* = 27 Mean ± SD	p value
Age (years)	30.0 (±4.8)	32.6 (±4.4)	0.041
Height (m)	1.6 (±0.1)	1.6 (±0.1)	0.724
Body weight (kg)	79.0 (±11.7)	77.3 (±9.6)	0.561
BMI at the booking visit (kg/m^2^)	32.3 (±5.5)	31.6 (±4.7)	0.596
Parity	1.4 (±0.4)	1.6 (±0.4)	0.104

### Outcomes and estimation

#### Primary outcome

The comparison of the mean post-operative pain scores at 48-h after CS between the intervention and the control groups revealed that the intervention group had obtained the least mean post-operative pain score compared to the control group (Table 3) .

**Table 3:** Comparison of the mean post-operative pain score at 48-h after CS between the intervention and the control groups

	Intervention group Mean ± SD	Control group Mean ± SD	Mean Difference	95% confidence intervals	p value
Post-operative pain score at 48-h after CS	1.7 ± 0.7	4.2 ± 0.4	2.5	2.1–2.8	< 0.001

#### Secondary outcomes

Participants of the intervention group obtained the least mean doses of the additional analgesics for the drugs Diclofenac Suppository, Paracetamol, Pethidine, and Tramadol compared to the control group (Table 4).

**Table 4:** Comparison of the doses of the additional analgesics required between the intervention and the control groups

	Intervention group Mean ± SD	Control group Mean ± SD	Mean Difference	95% confidence intervals	p value
Additional analgesics required					
1.Diclofenac Suppository	2.0 ± 0.0	2.2 ± 0.4	0.2	0.1–0.4	0.011
2.Paracetamol	3.5 ± 0.6	4.0 ± 0.0	0.5	0.2–0.7	< 0.001
3. Pethidine	0.0 ± 0.0	1.8 ± 1.3	1.8	1.3–2.3	< 0.001
4.Tramadol	0.0 ± 0.0	1.5 ± 1.3	1.5	1.0–2.0	< 0.001

In addition, the two groups differed significantly in terms of the lengths of hospital stay and the pain on returning to functional activities. (Table 5).

**Table 5:** Comparison of the mean lengths of hospital stay and pain upon returning to functional activities in post-natal life between the intervention and control groups

	Intervention group Mean ± SD	Control group Mean ± SD	Mean Difference	95% confidence intervals	p value
Lengths of hospital stay	3.0 ± 0.0	3.9 ± 0.3	0.9	0.7–1.0	< 0.001
Pain in returning to the functional activities in post-natal life - 1.Turning in bed without support					
Day 1	5.8 ± 0.4	7.7 ± 0.5	1.9	1.6–2.1	< 0.001
Day 2	3.3 ± 1.1	5.4 ± 0.7	2.1	1.6–2.6	< 0.001
2.Sitting without support					
Day 1	6.1 ± 0.4	7.7 ± 0.5	1.6	1.3–1.8	< 0.001
Day 2	4.1 ± 0.6	5.9 ± 0.5	1.8	1.4–2.0	< 0.001
3.Standing without support					
Day 1	6.5 ± 0.6	8.2 ± 0.6	1.7	1.3–1.9	< 0.001
Day 2	3.9 ± 0.4	6.1 ± 0.5	2.2	1.9–2.4	< 0.001
4.Walking without support					
Day 1	7.2 ± 0.6	8.5 ± 0.5	1.3	0.9–1.6	< 0.001
Day 2	4.0 ± 0.6	5.9 ± 0.6	1.9	1.5–2.1	< 0.001

No adverse events were observed in the study.

## Discussion

This is the first study done in Sri Lanka to assess the effectiveness of face-to-face physiotherapy training and education for women who are undergoing elective CS. This is the largest RCT so far documenting the effectiveness of physiotherapy exercises and education prior to elective CS to improve the post-operative outcomes. The results of the present study revealed that the face-to-face physiotherapy training and education prior to elective CS is a promising intervention to improve the post-operative outcomes by reducing post-operative pain, doses of additional analgesics required, pain upon returning to functional activities and lengths of hospital stay.

As we reach millennium development goals of reducing maternal mortality and morbidity, the focus throughout the world has drifted towards concepts such as holistic, humane, and respectful care of patients, among which pain relief is an important component. Pain relief is poorly addressed in Low-and Middle-Income Countries (LMIC). Knowledge and attitudes about pain management, low prioritization of pain management by governments and hospitals, inappropriate legislation, and limited or non-existent availability of pain treatments are some of the key issues existing in many settings. It is evident that advocacy, improving treatment availability, and education can improve the quality of pain management [30]. We believe that our interventions, based on the principles of physiotherapy and health education, would play a pivotal role in post-operative pain management and the overall quality of maternal health and wellbeing.

Breast feeding is perhaps the most important health intervention that has led to improvement in neonatal and childhood indicators of health and is an act that can lead to the development of a deep parental bond that continues to nurture our emotional lives into adulthood. However, CS has been detrimentally associated with lactation due to the pain and delayed return to functional activities encountered by lactating women [31–33]. Irrespective of the mode of delivery, all women, particularly in Asian countries including Sri Lanka, often play a major role in household activities, often caring for other children, aging parents and running households, and very few can afford to pay for these services [34]. They are inevitably placed in the role of carrying out these activities along with attending to and feeding a new born baby, despite the pain following surgery. Hence, there is a need to address pain and ensure an early return to functional activities to ensure that they may carry out the above-mentioned activities with little hindrance. A low-cost, evidence-based intervention may improve the above-mentioned activities.

Incisional care education and training to support the incision with a pillow when coughing or breast feeding have been shown to help reduce incision-related pain [2].Post-natal exercises, such as mobility exercises, breathing techniques, and postural care, were effective treatment strategies for post-operative incision pain [16]. This led to a significant reduction in the doses of additional analgesics required and the pain and difficulty in returning to functional activities in the intervention group compared to the control group. It has been found that early ambulation and return to normal pre-pregnancy physical activities and physiotherapy exercise routines as soon as medically stable could reduce co-morbidities associated with post-operative sedentary lifestyle and appear to enhance restoration of physical function after CS [35, 36].

Similar results were found in a study conducted by Karakaya et al. using transcutaneous electrical nerve stimulation as a part of physiotherapy management along with post-natal exercises. They stated that incision-related pain and difficulty in functional activities decreased significantly within 2 days in both groups, and the values were lower in the intervention group compared to those of the control group. In fact, the study group needed less medication for pain control. They emphasized the fact that the intervention group showed a rapid post-operative recovery as they had shown significantly lower difficulty during the functional activities such as turning in bed, sitting, standing, and walking, according to VAS, than the control group within 2 days following CS [15].

These findings correlate favorably with the study conducted by Ul Ain et al. on the ease of pain and functional activities following delivery after post-natal exercises. They reported higher mean post-operative incision related pain scores and mean pain scores in returning to the same set of functional activities by the control group, compared to the intervention group [16]. It is encouraging to compare these findings with that found by Burti et al., who showed that the exercise protocol optimistically contributed to the reduction of pain and improvement of general well-being [22]. Breathing exercises have been shown to improve circulation and healing when combined with mild abdominal muscle activity [15].

Although we found that there was a significant difference in the lengths of hospital stay between the intervention and the control groups, the results of the study by Hollinger et al. suggest that the lengths of hospital stay of women using transcutaneous electrical nerve stimulation were not significantly different than those who did not use it [8]. This inconsistency may be due to the difference in the particular physiotherapy intervention used between the above study and the present study. Since they had adopted transcutaneous electrical nerve stimulation as the treatment modality, which serves only a pain-relieving purpose, there could be a discrepancy between the results obtained from the two studies. The inclusion of exercises and education guidelines in the current study’s treatment protocol to improve pulmonary function and reduce the risk of pneumonia, decrease incision-related pain with coughing, movements, or breastfeeding, prevent post-surgical vascular or gastrointestinal complications, improve incision circulation and healing, and prevent adhesion formation and posture correction has resulted in a significant reduction in hospitalization duration. There are socio-cultural differences among different nations and even within particular healthcare institutions. Since other studies that have assessed the effectiveness of physiotherapy training and education prior to elective CS to improve the post-operative outcomes have not focused their attention on the effectiveness of physiotherapy in reducing the lengths of hospital stays, we suggest that further research on the effect of physiotherapy on hospital stays and costs involved would be more useful in informing policy makers to strengthen existing physiotherapy services in the country.

### Generalizability and limitations

To the best of our knowledge, this is the first RCT performed in a LMIC to address the importance of physiotherapy for improving post-operative outcomes following CS.

The generalizability of these results is subject to certain limitations. As this was a single blind RCT, the investigators were aware of the control and intervention arms.

Since the participant allocation for the intervention and the control arms was random, there is hardly any chance of introducing bias at this point [37]. The ascertainment of outcomes was also done through hospital records (doses of additional analgesics required and lengths of hospital stay) or self-reporting. Therefore, there is hardly any chance of introducing any bias into the ascertainment of outcomes. However, reporting bias cannot be completely excluded as the mothers who received the intervention had more frequent and close contact with the physiotherapist and tended to report more positive outcomes than the control arm. And this could have led to a certain amount of ascertainment bias during the data collection.

The selection of the study population for this study was limited to only one hospital because this hospital treats pregnant mothers from many districts of the country, and a sufficient number of mothers who were to undergo elective CS can be recruited from this hospital. As the study sample was selected in a single setting, the results may be biased and may not represent the entire population of mothers who have undergone elective CS in Sri Lanka. As this is a centre of excellence in maternity care in Sri Lanka, the delivery of the intervention and its quality would have been exceptional. This may have led to a greater impact than expected.

Since the VAS was used to assess pain in the post-op period, there is likely to be an element of subjectivity in individual pain assessment, which may impact the final scores. The size of the subject population in the study is a major constraint on the generalization of the study results. Hence, further studies may be needed to assess the broader applicability of such a study along with variations in the duration, repetitions, and frequency of the exercises to identify the optimal exercise prescription needed to reduce post-operative morbidity.

## Conclusion

The results of this RCT suggest that face-to-face physiotherapy training and education prior to elective CS is effective in improving the post-operative outcomes by reducing the post-operative pain score, the need of additional analgesics, the pain in returning to functional activities in post-natal life, and the lengths of hospital stay. Further research on the impact on long-term implications and economic evaluations of face-to-face physiotherapy training and education prior to elective CS would be useful in informing policymakers.

## Data Availability

The datasets used and/or analyzed during the current study are available from the corresponding author on reasonable request.

## Abbreviations

CS: Caesarean Section
RCT: Randomized Controlled Trial
VAS: Visual Analogue Scale
DSHW: De Soysa Hospital for Women
DMARDs: Disease Modifying Anti Rheumatic Drugs
ICU: Intensive Care Unit
DRA: Diastasis Rectus abdominis
SD: Standard Deviation
BMI: Body Mass Index
LMIC: Low-and Middle-Income Countries
